# Criteria for robustness of heteroclinic cycles in neural microcircuits

**DOI:** 10.1186/2190-8567-1-13

**Published:** 2011-11-28

**Authors:** Peter Ashwin, Özkan Karabacak, Thomas Nowotny

**Affiliations:** 1 Mathematics Research Institute, University of Exeter, Exeter, EX4 4QF, UK; 2 Faculty of Electrical and Electronics Engineering, Electronics and Communication Department, Istanbul Technical University, TR-34469, Maslak-Istanbul, Turkey; 3 Centre for Computational Neuroscience and Robotics, Informatics, University of Sussex, Falmer, Brighton, BN1 9QJ, UK

## Abstract

We introduce a test for robustness of heteroclinic cycles that appear in neural microcircuits modeled as coupled dynamical cells. Robust heteroclinic cycles (RHCs) can appear as robust attractors in Lotka-Volterra-type winnerless competition (WLC) models as well as in more general coupled and/or symmetric systems. It has been previously suggested that RHCs may be relevant to a range of neural activities, from encoding and binding to spatio-temporal sequence generation.

The robustness or otherwise of such cycles depends both on the coupling structure and the internal structure of the neurons. We verify that robust heteroclinic cycles can appear in systems of three identical cells, but only if we require perturbations to preserve some invariant subspaces for the individual cells. On the other hand, heteroclinic attractors can appear robustly in systems of four or more identical cells for some symmetric coupling patterns, without restriction on the internal dynamics of the cells.

## 1 Introduction

 For some time, it has been recognized that robust heteroclinic cycles (RHCs) can be attractors in dynamical systems [1], and that RHCs can provide useful models for the dynamics in certain biological systems. Examples include Lotka-Volterra population models [2] in ecology and game dynamics [3]. Similar dynamics has been used to describe various neuronal microcircuits, in particular winnerless competition (WLC) dynamics [4] has been the subject of intense recent study. For example, [5] find conditions on the connectivity scheme of the generalised Lotka-Volterra model to guarantee the existence and structural robustness of a heteroclinic cycle in the system, [6] consider generalised “heteroclinic channels”, [7] use them as a model for sequential memory and [8] suggest that they may be used to describe binding problems. One question raised by these studies is whether Lotka-Volterra type dynamics is necessary to give robust heteroclinic cycles as attractors and how these cycles relate to those found in other models [9,10]. The purpose of this paper is to show that attracting heteroclinic cycles may be robust for a variety of reasons and appear in a variety of dynamical systems that model neural microcircuits. In doing so, we give a practical test for robustness of heteroclinic cycles within any particular context and demonstrate it in practice for several examples.

 This paper was motivated by a recent paper on three synaptically coupled Hodgkin-Huxley type neurons in a ring that reported robust winnerless competition between neurons [11] without an explicit Lotka-Volterra type structure. This manifested as a cyclic progression between states where only one neuron is active (spiking) for a period of time. During this activity, the currently active neuron inhibits the activity of the next neuron in the ring while the third neuron recovers from previous inhibition.

 One of the main observations of this paper is that the coupling structure and symmetries in this system are not sufficient to guarantee robustness of the heteroclinic behaviour observed in [11], but robustness can be demonstrated if we consider *constraints* in the system. For this case it is natural to investigate the invariance of a set of affine subspaces of the system’s phase space related to the type of synaptic coupling considered. More generally, we discuss cases of heteroclinic attractors that are robust, based purely on the coupling structure and the assumption that the cells are identical.

 The paper is organized as follows: In Section 2 we consider the general problem of robustness of a heteroclinic cycle. We investigate a class of dynamical systems that have affine invariant subspaces and give a necessary and sufficient condition on the dimensionality of the invariant affine subspaces for the robustness of HCs in this class of systems. We translate these conditions into appropriate conditions for coupled systems. Section 3.1 reviews a simple example of winnerless competition and demonstrates robustness for Lotka-Volterra systems, while Section 3.2 discusses the three-cell problem of Nowotny *et al.*[11]. We demonstrate how the general results from Section 2 can be applied to show that the observed HC in the system (i) is not robust with respect to perturbations that only preserve its $Z_{3}$ symmetry, but (ii) is robust with respect to perturbations that respect a specific set of invariant affine subspaces. Section 3.3 illustrates an example of a four-cell network of Hodgkin-Huxley type neurons where the coupling structure alone is sufficient for the robustness of HCs. We finish with a brief discussion in Section 4.

## 2 Robustness of heteroclinic cycles

Suppose we have a dynamical system given by a system of first order differential equations

(1) $$\frac{dx}{dt}=f(x)$$

where $x\in R^{n}$ and f∈X, the set of $C^{1}$ vector fields on $R^{n}$ with bounded global attractors.^1^ We say an invariant set Σ is a *heteroclinic cycle* (HC) if it consists of a union of hyperbolic equilibria $\left\{x_{i}:i=1,\ldots ,p\right\}$ and connecting orbits $s_{i}\subset W^{u}(x_{i})\cap W^{s}(x_{i+1})$.^2^ We say that a heteroclinic cycle Σ is *robust to perturbations in*Y⊂X if f∈Y and there is a $C^{1}$-neighbourhood of *f* such that all g∈Y within this neighbourhood have a heteroclinic cycle that is close to Σ.

 Let us suppose that f∈X has a HC Σ between equilibria $x_{i}$. As the connection $s_{i}$ is contained within $W^{u}(x_{i})\cap W^{s}(x_{i+1})$, this implies that $\dim(W^{u}(x_{i})\cap W^{s}(x_{i+1}))\geq 1$. In order for the connection from $x_{i}$ to $x_{i+1}$ to be robust with respect to arbitrary $C^{1}$ perturbations it is necessary that the intersection is transverse [12], meaning that

(2) $$\dim(W^{u}(x_{i}))+\dim(W^{s}(x_{i+1}))\geq n+1.$$

Using the fact that $\dim(W^{u}(x_{i}))+\dim(W^{s}(x_{i}))=n$ for any hyperbolic equilibrium and adding these for all equilibria along the cycle, we find that

(3) $$\sum_{i=1}^{p}\left[\dim(W^{u}(x_{i}))+\dim(W^{s}(x_{i+1}))\right]=pn.$$

This implies that it is not possible for Equation 2 to be satisfied for all connections. Hence our first statement is the following (which can be thought of a special case of the Kupka-Smale Theorem [12], see also [13]).

**Proposition 1***A heteroclinic cycle between*p>0*hyperbolic equilibria is never robust to general*$C^{1}$*perturbations in*X.

The heteroclinic cycle may however be robust to a constrained set of perturbations. We explore this in the following sections.

### 2.1 Conditions for robustness of heteroclinic cycles with constraints

A subset $I\subset R^{n}$ is an *affine subspace* if it can be written as $I:=\{x\in R^{n}:Ax=b\}$ for some real-valued n×n matrix *A* and vector $b\in R^{n}$ (this is a linear subspace if *b* can be chosen to be zero). For a given phase space $R^{n}$, suppose that we have a (finite) set of non-empty affine subspaces

(4) $$I=\{I_{1},\ldots ,I_{d}\}$$

that are closed under intersection; i.e. the intersection $I_{j}\cap I_{k}$ of any two subspaces $I_{j},I_{k}\in I$ is an element of I unless it is empty. We include $I_{1}=R^{n}$, which is trivially invariant, so I is always non-empty. For a given I, we define the set of *vector fields (in*X*) respecting*I to be

(5) $$X_{I}:=\{f\in X:f(I)\subset I\text{ for all }I\in I\}$$

and call the subspaces in I*invariant* subspaces in the phase space of the dynamical systems described by $f\in X_{I}$.

 A set of invariant affine subspaces I may arise from a variety of modelling assumptions; for example, 

• If *f* is a Lotka-Volterra type population model that leaves some subspaces corresponding to the absence of one or more “species” invariant then $f\in X_{I}$ where I is the set of the invariant subspaces forced by the absence of these species.

• If *f* is symmetric (equivariant) for some group action *G* and I is the set of fixed point subspaces of *G* then $f\in X_{I}$ because fixed point subspaces are invariant under the dynamics of equivariant systems [14], Theorem 1.17]. Note that for an orthogonal group action, the fixed point subspaces are linear subspaces. It is known that symmetries impose further constraints on the dynamics such as repeated eigenvalues or missing terms in Taylor expansions [14] but we focus here only on the invariant subspaces.

• If *f* is a realization of a particular coupled cell system with a given coupling structure then $f\in X_{I}$ where I corresponds to the set of possible cluster states (also called synchrony subspaces or polydiagonals in the literature [15-17]).

 Note that $X_{I}$ inherits a subset topology from X; for a discussion of homoclinic and heteroclinic phenomena in general and their associated bifurcations in particular, we refer to the review [13].

Suppose that for a vector field $f\in X_{I}$ we have a heteroclinic cycle Σ between hyperbolic equilibria $\left\{x_{i}\right\}$ (i=1,…,p) with connections $s_{i}$ from $x_{i}$ to $x_{i+1}$. We define

(6) $$I_{c(i)}:=\underset{\left\{c:s_{i}\subset I_{c}\in I\right\}}{⋂}I_{c}$$

i.e. the smallest subspace in I that contains $s_{i}$. The invariant set $I_{c(i)}$ is clearly well defined because I is closed under intersections. We define the *connection scheme* of the heteroclinic cycle to be the sequence

(7) $$x_{1}\overset{I_{c(1)}}{\to }x_{2}\overset{I_{c(2)}}{\to }\cdots \overset{I_{c(p)}}{\to }x_{1}.$$

The following theorem gives necessary and sufficient conditions for such a heteroclinic cycle to be robust to perturbations in $X_{I}$, depending on its connection scheme (we will require robustness to preserve the connection scheme). More precisely it depends on the following equation being satisfied:

(8) $$\dim(W^{u}(x_{i})\cap I_{c(i)})+\dim(W^{s}(x_{i+1})\cap I_{c(i)})\geq \dim(I_{c(i)})+1$$

for each *i*. Note that there is a slight complication for the sufficient condition - it may be necessary to perturb the system slightly within $X_{I}$ to unfold the intersection to general position and remove a tangency between $W^{u}(x_{i})$ and $W^{s}(x_{i+1})$. *This complication has the benefit that it allows us to make statements about particular connections without needing to verify that the intersection of manifolds is transverse.*

**Theorem 1***Let* Σ *be a heteroclinic cycle for*$f\in X_{I}$*between hyperbolic equilibria*$\left\{x_{i}:i=1,\ldots ,p\right\}$*with connection scheme Equation *7. 

1. *If the cycle* Σ *is robust to perturbations in*$X_{I}$*then Equation *8*is satisfied for*i=1,…,p.

2. *Conversely*, *if Equation *8*is satisfied for*i=1,…,p*then there is a nearby*$\tilde{f}\in X_{I}$ (*with*$\tilde{f}$*arbitrarily close tof*) *such that* Σ *is a heteroclinic cycle for*$\tilde{f}$*that is robust to perturbations in*$X_{I}$.

*Proof* We will abbreviate $I_{c}:=I_{c(i)}$. Because $s_{i}$ is a connection from $x_{i}$ to $x_{i+1}$, there is a non-trivial intersection of $W^{u}(x_{i})\cap W^{s}(x_{i+1})$ within $I_{c}$. As $I_{c}$ is the smallest invariant subspace containing $s_{i}$, typical points $y\in s_{i}$ will have a neighbourhood in $I_{c}$ that contain no points in any other $I_{j}$. In a neighbourhood of this *y*, perturbations of *f* in $X_{I}$ have no restriction other than they should leave $I_{c}$ invariant.

 The stability of the intersection of the unstable and stable manifolds depends on the dimension of the unstable manifolds (also called the *Morse index*[13]) for these equilibria for the vector field restricted to $I_{c}$. Pick any codimension one section $P\subset I_{c}$ transverse to the connection at *y*. We have

(9) $$\dim(P)=\dim(I_{c})-1$$

and within *P*, the invariant manifolds have dimensions

(10) $$\begin{array}{rcl} \dim(W^{u}(x_{i})\cap P) & = & \dim(W^{u}(x_{i})\cap I_{c})-1,\\ \dim(W^{s}(x_{i+1})\cap P) & = & \dim(W^{s}(x_{i+1})\cap I_{c})-1. \end{array}$$

The intersection of these invariant manifolds may not be transverse within *P*, but it will be for a dense set of nearby vector fields. In particular, if

(11) $$\dim(W^{u}(x_{i})\cap P)+\dim(W^{s}(x_{i+1})\cap P)<\dim(P)$$

then there will be an open dense set of perturbations of *f* that remove the intersection, giving lack of robustness of $s_{i}$ and hence we obtain a proof for case 1. On the other hand, if Equation 11 is not satisfied, we can choose a vector field $\tilde{f}$ that is identical to *f* except on a small neighbourhood *ofy- there it is chosen to preserve the connection but to perturb the manifolds so that the intersection is transverse.* Transversality of the intersection then implies robustness of the connection and hence we obtain a proof for case 2. □

Note that caution is necessary in interpreting this result for a number of reasons: 

1. Just because a *given* heteroclinic connection is not robust due to this result does not necessarily imply that there is no robust connection from $x_{i}$ to $x_{i+1}$ at all. Indeed, it may be [18] that there are several connections from $x_{i}$ to $x_{i+1}$ and that perturbations will break some but not all of them. In this sense, it may be that at the same time, one heteroclinic cycle is not robust, but another heteroclinic cycle between the same equilibria is robust.

2. We consider robustness to perturbations that preserve the connection scheme - there are situations where a typical perturbation may break a connection but preserve a nearby connection in a larger invariant subspace. This situation will typically only occur in exceptional cases.

3. The structure of general robust heteroclinic cycles may be very complex even if we only examine cases forced by symmetries - they easily form networks with multiple cycles. There may be multiple or even a continuum of connections between two equilibria, and they may be embedded in more general “heteroclinic networks” where there may be connections to “heteroclinic subcycles” [16,19,20].

4. Theorem 1 does not consider any dynamical stability (attraction) properties of the heteroclinic cycles.

5. In what follows, we slightly abuse notation by saying that a heteroclinic cycle is robust if the cycle for an arbitrarily small perturbation of the vector field is robust.

 If $W^{u}(x_{i})$ is not contained in $W^{s}(x_{i+1})$ then the heteroclinic cycle Σ cannot be asymptotically stable. We say that an invariant set Σ is a *regular heteroclinic cycle* if it consists of a union of equilibria and a set of connecting orbits $s_{i}\subset W^{u}(x_{i})$ with $W^{u}(x_{i})\subset W^{s}(x_{i+1})$. The following result is stated in [13] for the case of symmetric systems.

**Theorem 2***Suppose that* Σ *is a regular heteroclinic cycle for*$f\in X_{I}$*between hyperbolic equilibria*$\left\{x_{i}:i=1,\ldots ,p\right\}$. *Suppose that*$x_{i+1}$*is a sink for the dynamics reduced to*$I_{c(i)}$, *i*.*e*.

(12) $$\dim(W^{s}(x_{i+1})\cap I_{c(i)})=\dim(I_{c(i)})$$

*for alli*. *Then the heteroclinic cycle is robust to perturbations within*$X_{I}$.

*Proof* Suppose that $W^{s}(x_{i+1})⊃I_{c(i)}$. Since $W^{u}(x_{i})$ is contained in $W^{s}(x_{i+1})$ by regularity of the HC, and because $I_{c(i)}⊇s_{i}=W^{u}(x_{i})$, we find $\dim(W^{u}(x_{i})\cap I_{c(i)})+\dim(W^{s}(x_{i+1})\cap I_{c(i)})=\dim(W^{u}(x_{i}))+\dim(I_{c(i)})\geq \dim(I_{c(i)})+1$. Hence, Equation 8 follows and we could apply Theorem 1 case 2. In fact this is a simpler case in that because $\dim(W^{s}(x_{i+1})\cap I_{c(i)})=\dim(I_{c(i)})$ the intersection must already be transverse - one does not need to consider any perturbations to force transversality of the intersection. □

### 2.2 Cluster states for coupled systems

 RHCs may appear in coupled systems due to a variety of constraints from the coupling structure - these are associated with cluster states (also called synchrony subspaces [15] or polydiagonals for the network [21]). Consider a network of *N* systems each with phase space $R^{d}$ and coupled to each other to give a set of differential equations on $R^{n}$, with n=Nd, of the form

(13) $$\frac{dx_{i}}{dt}=f_{i}(x_{1},\ldots ,x_{N})$$

for $x_{i}\in R^{d}$i=1,…,N. We write $f:R^{Nd}\to R^{Nd}$ with $f(x)=(f_{1}(x),\ldots ,f_{N}(x))$. We define a *cluster state* for a class of ODEs to be a partition $P_{i}$ of {1,…,N} such that the linear subspace

$$I_{i}:=\{(x_{1},\ldots ,x_{N}):x_{j}=x_{k}\Leftrightarrow \{j,k\}\text{ are in the same part of }P_{i}\}$$

is dynamically invariant for all ODEs in that class. For a given symmetry or coupling structure, we identify a list of possible cluster states and use these to test for robustness of any given heteroclinic cycle using Theorem 1.

 We remark that the simplest (and indeed only, up to relabelling) coupling structure for a network of three identical cells found by [15] to admit heteroclinic cycles can be represented as a system of the form

(14) $$\begin{array}{rcl} \dot{x} & = & f(x;y,z),\\ \dot{y} & = & f(y;x,z),\\ \dot{z} & = & f(z;y,x). \end{array}$$

For an open set of choices of f(x,y,z), the heteroclinic cycle involves two saddles within the subspace $I_{1}:=\{x=y=z\}$ and connections that are contained within $I_{2}:=\{x=y\}$ in one direction and $I_{3}:=\{x=z\}$ in the other. This represents a system of three identical units coupled in a specific way, where each unit has two different input types; we refer to [15] for details. It can be quite difficult to find a suitable function *f* that gives a robust heteroclinic cycle in this case. Nevertheless, once one has found a heteroclinic cycle, it can be shown to be robust using Theorem 1 (case 2).

 Other examples of robust heteroclinic cycles between equilibria for systems of coupled phase oscillators are given in [22,23]. For such systems the final state equations are obtained by reducing the dynamics to phase difference variables. In this case, each equilibrium represents the oscillatory motion of oscillators with some fixed phase difference.

### 2.3 Robust heteroclinic cycles between periodic orbits

In cases where a phase difference reduction is not possible, one may need to study heteroclinic cycles between periodic orbits in order to explain heteroclinic behaviour. Unlike heteroclinic cycles between equilibria, heteroclinic cycles between periodic orbits can be robust under general perturbations since for a hyperbolic periodic orbit *p*, $\dim(W^{u}(p))+\dim(W^{s}(p))=n+1$. Hence, the condition Equation 2 can be satisfied. For instance, consider a system on $R^{3}$ with two hyperbolic periodic orbits *p* and *q* for which the stable and unstable manifolds $W^{s}(p)$, $W^{u}(p)$, $W^{s}(q)$, and $W^{u}(q)$ are two-dimensional. In this case, $W^{u}(p)$ and $W^{s}(q)$ (and similarly, $W^{u}(q)$ and $W^{s}(p)$) intersect transversely, and therefore, a heteroclinic cycle between *p* and *q* can exist robustly. However, for this heteroclinic cycle only one orbit connects *p* to *q*, whereas infinitely many orbits which are backward asymptotic to *p* move away from the heteroclinic cycle. As a result, such a robust heteroclinic cycle cannot be asymptotically stable.

To overcome this difficulty we assume that the connections of a heteroclinic cycle between periodic orbits consist of unstable manifolds of periodic orbits and these are contained in the stable manifold of the next periodic orbit. Namely, we say an invariant set Σ is a *heteroclinic cycle that contains all unstable manifolds* if it consists of a union of periodic orbits and/or equilibria $\left\{x_{i}:i=1,\ldots ,p\right\}$ and a set of connecting manifolds $S_{i}=W^{u}(x_{i})$ with $W^{u}(x_{i})\subset W^{s}(x_{i+1})$.

**Theorem 3***Suppose that* Σ *is a heteroclinic cycle that contains all unstable manifolds for*$f\in X_{I}$*between hyperbolic equilibria or periodic orbits*$\left\{x_{i}:i=1,\ldots ,p\right\}$. *If there exists a finite sequence*$\left\{I_{c(1)},\ldots ,I_{c(p)}\right\}$*of elements in*I*such that*$I_{c(i)}⊃S_{i}$*and*

(15) $$\dim(W^{s}(x_{i+1})\cap I_{c(i)})=\dim(I_{c(i)})$$

(*in other words*, $x_{i+1}$*is a sink for the dynamics reduced to*$I_{c(i)}$) *for all*i=1,…,p*then* Σ *is robust to perturbations within*$X_{I}$.

*Proof* Consider a unique orbit $s_{i}\subset S_{i}$. Since $W^{s}(x_{i+1})$*contains a neighbourhood of*$x_{i+1}$*in*$I_{c(i)}$, $s_{i}$ is robust by the same reasoning as in the proof of Theorem 2. This implies that the manifold of connections $S_{i}$ is robust for all *i*. □

 Note that a heteroclinic cycle may contain all unstable manifolds but not be attracting even in a very weak sense (essentially asymptotically stable [24]). Conversely, a heteroclinic cycle may not contain all unstable manifolds but may be essentially asymptotically stable.

## 3 Robust heteroclinic behaviour in neural models

We discuss three examples of cases where robust heteroclinic behaviour can be found in simple neural microcircuits.

### 3.1 Winnerless competition in Lotka-Volterra rate models

 The review [25] includes a discussion of winnerless competition and related phenomena. This has focused on the dynamics of Lotka-Volterra type models for firing rates, justified by an approximation of Fukai and Tanaka [26]. In their most general form, these are written as

(16) $${\dot{x}}_{i}=x_{i}F_{i}(x)$$

where $x_{i}$ for i=1,…,N is the firing rate of some neuron (or neural assembly) and $F_{i}(x)$ is a nonlinear function that represents both the intrinsic firing and that due to interaction with the other cells in the network. These systems have a very rich set of invariant subspaces because of the invariance of all subspaces where $x_{i}=0$. More precisely, given any subset S⊂{1,…,N} there is an invariant subspace corresponding to

$$I_{S}:=\{x:x_{i}=0\text{ if }i\in S\};$$

for example $I_{\left\{2,4\right\}}:=\{x:x_{2}=x_{4}=0\}$. This gives a total of $2^{N}$ invariant subspaces for the dynamics of Equation 16. Using these one can find a connection scheme involving these $I_{S}$ such that Theorem 1 can be applied to check robustness of a specific heteroclinic cycle to perturbations that preserve the form Equation 16. For example, the following rate model for the pyloric CPG of the lobster stomatogastric ganglion is discussed in [25]:

(17) $$\frac{da_{i}(t)}{dt}=a_{i}(t)\left(1-\sum_{j=1}^{N}\rho_{ij}(S_{i})a_{j}(t)\right)+S_{i}$$

with $S_{i}$ representing the stimulus and $a_{i}(t)$ the firing rate of the *i*th neuron. In the absence of stimulus $S_{i}=0$ this exhibits heteroclinic cycles for N=3

$$\rho (0)=(\begin{array}{ccc} 1 & 1.25 & 0\\ 0.875 & 1 & 1.25\\ X/80 & 0.625 & 1 \end{array})$$

and X>160. These heteroclinic cycles connect three equilibria of Equation 17, namely $x_{1}=(1,0,0)\to x_{2}=(0,1,0)\to x_{3}=(0,0,1)$. Calculating linearizations of Equation 17 at these equilibria one can show that, for the equilibrium $x_{i}$, three linearly independent eigenvectors are contained in $I_{\left\{j,k\right\}}$$I_{j}$ and $I_{k}$ with eigenvalues $1-2\rho_{ii}$$1-\rho_{ji}$$1-\rho_{ki}$, respectively, where i,j,k∈{1,2,3} are different indices. Hence, when *ρ* is chosen as above, it follows that

$$\begin{array}{rcl} \dim(W^{u}(x_{1})\cap I_{3})+\dim(W^{s}(x_{2})\cap I_{3})=3 & \geq & \dim(I_{3})+1=3\\ \dim(W^{u}(x_{2})\cap I_{1})+\dim(W^{s}(x_{3})\cap I_{1})=3 & \geq & \dim(I_{1})+1=3\\ \dim(W^{u}(x_{3})\cap I_{2})+\dim(W^{s}(x_{1})\cap I_{2})=3 & \geq & \dim(I_{2})+1=3. \end{array}$$

Finally, from Theorem 1 case 2, we can conclude that the heteroclinic cycle between saddle equilibria $x_{1}\in I_{\left\{2,3\right\}}$$x_{2}\in I_{\left\{1,3\right\}}$ and $x_{3}\in I_{\left\{1,2\right\}}$ is robust for the robust connection scheme

$$x_{1}\overset{I_{\left\{3\right\}}}{\to }x_{2}\overset{I_{\left\{1\right\}}}{\to }x_{3}\overset{I_{\left\{2\right\}}}{\to }x_{1}.$$

### 3.2 Robustness of a heteroclinic cycle in a rate model with synaptic coupling

 We now turn to the robustness of heteroclinic cycles in a specific model of N=3 coupled neurons derived from a Hodgkin-Huxley type model with synaptic coupling [11], a case where we do not have the Lotka-Volterra structure Equation 16. If the synaptic time scales are slow compared to the time scale of the individual spikes, then the full conductance based model can be reduced systematically to an approximate rate model [11], equations (13,14)]:

(18) $$\begin{array}{rcl} \tau \frac{ds_{i}}{dt} & = & \left(r_{i}-\frac{s_{i}}{2}\right)\frac{S_{\max}-s_{i}}{S_{\max}}\\ \tau \frac{dr_{i}}{dt} & = & x_{0}F\left(I-\sum_{j=1}^{N}g_{ij}s_{j}\right)\tau -r_{i}, \end{array}$$

where time variable *t* is in ms. The unitless dynamical variables $r_{i}$ represent the fraction of presynaptically released and $s_{i}$ the fraction of postsynaptically bound neurotransmitter for the *i*th neuron (i=1,…,N), and

$$F(x)=\exp(-\epsilon /x){\left(\max(0,x)\right)}^{\alpha }$$

characterises the rate response of the neurons to input current. We have introduced a smoothing factor exp(−ϵ/x), with small ϵ>0 to ensure that *F* is $C^{1}$ without affecting the overall structure of the model. We use parameters as in Table 1 and couple N=3 cells in a ring using different coupling strength in each direction:

(19) $$\begin{array}{rcl} g_{21} & = & g_{32}=g_{13}=g_{1},\;g_{12}=g_{23}=g_{31}=g_{2},\\ g_{11} & = & g_{22}=g_{33}=0. \end{array}$$

**Fig. 1:**
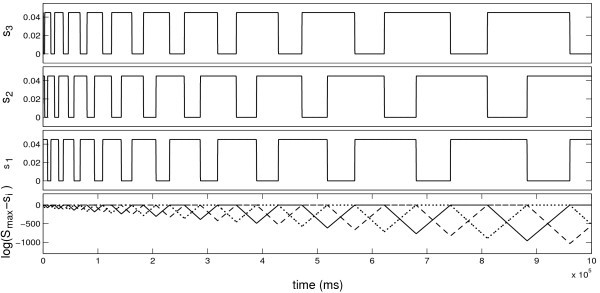
A trajectory approaching a heteroclinic cycle for the rate model (18) with $\epsilon =10^{-3}$ and parameters as in Table 1. Observe that the trajectory cycles between the neighbourhoods of three saddle equilibria where two of the three $s_{i}$ are close to saturated at $S_{\max}=0.045$. The switching dynamics between equilibria continues to slow down indicating that the trajectory is approaching the actual heteroclinic orbit. The bottom panel illustrates this further by showing that the $s_{i}$ continue to approach the equilibria over the whole duration of the simulation. The simulation is performed using a numerical scheme that carefully resolves the behaviour near the equilibria.

A typical timeseries showing an attracting heteroclinic cycle for this system is shown in Figure 1. 

**Table 1:** Numerical values of parameters used for simulation of the rate model (18); see [11] for a discussion of the derivation of the model and the meaning of the parameters.

Parameter	Value
*τ*	50 ms
*I*	0.145
$g_{1}$	3
$g_{2}$	0.7
$S_{\max}$	0.045
$x_{0}$	2.57 × 10^−3^ kHz
*α*	0.564

The heteroclinic cycle $x_{1}\to x_{3}\to x_{2}\to x_{1}$ connects the saddle equilibria $x_{1}$, $x_{2}$, $x_{3}$ listed in Table 2, all of which have one-dimensional unstable manifolds (unstable eigenvalue 0.0062) and five-dimensional stable manifolds (stable eigenvalues −0.0066, −0.01, −0.02, −0.02, −0.02). Adjusting any of these parameters appears to preserve the heteroclinic attractor. This raises the question whether the symmetry in the system is necessary or sufficient to ensure robustness of a heteroclinic cycle. We investigate the robustness of this cycle in the light of Theorem 1 to show that in fact the presence of this symmetry is neither necessary nor sufficient to ensure robustness. 

**Table 2:** Equilibria of (18) involved in the heteroclinic cycle for $\epsilon =10^{-3}$ and parameters as in Table 1.

	$r_{1}$	$s_{1}$	$r_{2}$	$s_{2}$	$r_{3}$	$s_{3}$	$W^{u}$ contained in
$x_{1}$	0	0	0.00866	$S_{\max}$	0.03733	$S_{\max}$	$s_{2}=S_{\max}$
$x_{2}$	0.03733	$S_{\max}$	0	0	0.00866	$S_{\max}$	$s_{3}=S_{\max}$
$x_{3}$	0.00866	$S_{\max}$	0.03733	$S_{\max}$	0	0	$s_{1}=S_{\max}$

**Theorem 4***There are heteroclinic cycles in the system Equation *18*with parameters in Table *1. *These cycles*: 

• *are not robust to perturbations that preserve the*$Z_{3}$*symmetry of cyclic permutation of the cells*.

• *are robust to perturbations that preserve the affine subspaces associated with*$s_{i}=S_{\max}$.

*Proof(We do not rigorously prove that the heteroclinic cycles exists; this should in principle be possible via rigorous methods with an error bounded integrator - see for example*[27]*.)* To show the first part, note that the only invariant subspaces in $\left(r_{1},s_{1},r_{2},s_{2},r_{3},s_{3}\right)$ permitted by $Z_{3}$ permutation symmetries are

$$I_{1}:=R^{6},\;I_{2}:=\{(r,s,r,s,r,s):(r,s)\in R^{2}\}.$$

Since c(i)=1 in all cases, Theorem 1 case 1 implies that typical symmetry-preserving perturbations of the system destroy the heteroclinic cycle.

To see the second part, let us consider the set of vector fields on $R^{6}$ that preserve the property that $s_{i}=S_{\max}$ is invariant: this means that we assume that the following set of subspaces are invariant:

(20) $$\begin{array}{rcl} I_{1} & := & R^{6},\;I_{5}:=\{x\in R^{6}:s_{1}=s_{2}=S_{\max}\},\\ I_{2} & := & \{x\in R^{6}:s_{1}=S_{\max}\},\;I_{6}:=\{x\in R^{6}:s_{2}=s_{3}=S_{\max}\},\\ I_{3} & := & \{x\in R^{6}:s_{2}=S_{\max}\},\;I_{7}:=\{x\in R^{6}:s_{3}=s_{1}=S_{\max}\},\\ I_{4} & := & \{x\in R^{6}:s_{3}=S_{\max}\},\;I_{8}:=\{x\in R^{6}:s_{1}=s_{2}=s_{3}=S_{\max}\}. \end{array}$$

Examining the equilibria in Table 2 we note that the $x_{i}$ are connected in the following connection scheme:

$$x_{1}\overset{I_{3}}{\to }x_{3}\overset{I_{2}}{\to }x_{2}\overset{I_{4}}{\to }x_{1}.$$

For the particular choice of parameters in Table 1, there is a heteroclinic cycle between three equilibria $x_{1}\in I_{6}$, $x_{2}\in I_{7}$, $x_{3}\in I_{5}$. These equilibria have unstable/stable manifolds that intersect to form a heteroclinic loop and satisfy

$$\begin{array}{rcl} \dim(W^{u}(x_{1})\cap I_{3})+\dim(W^{s}(x_{3})\cap I_{3})=6 & \geq & \dim(I_{3})+1=6\\ \dim(W^{u}(x_{3})\cap I_{2})+\dim(W^{s}(x_{2})\cap I_{2})=6 & \geq & \dim(I_{2})+1=6\\ \dim(W^{u}(x_{2})\cap I_{4})+\dim(W^{s}(x_{1})\cap I_{4})=6 & \geq & \dim(I_{4})+1=6. \end{array}$$

Hence the criteria of Theorem 1 (case 2) are satisfied and the heteroclinic cycle is robust with respect to $C^{1}$-perturbations that preserve the subspaces Equation 20. □

### 3.3 Robustness of heteroclinic cycles for a delay-coupled Hodgkin-Huxley type model

 One might suspect that Theorem 4 can be generalized to show that internal constraints might be needed to give robustness of HCs for larger numbers of cells, but this is not the case as long as the cells are assumed identical. For example [28-30] find robust cycles in systems of four or more identical, globally coupled phase oscillators with no further constraints.

 To illustrate this, we give an example of a robust heteroclinic attractor for a model system of four synaptically coupled neurons. We use a modification of Rinzel’s neuron model [31] presented by Rubin [32] with synaptic coupling [32]. Due to the global coupling of the system, the invariant subspaces are all nontrivial cluster states.

Consider *N* all-to-all synaptically coupled neurons with delay coupling (using units of mV for voltages, ms for time, mS/cm^2^ for conductances, *μ*A/cm^2^ for currents, and *μ*F/cm^2^ for capacitance):

(21) $$\begin{array}{rcl} c{\dot{v}}_{i}(t) & = & I_{i}-g_{L}(v_{i}(t)-v_{L})-g_{K}n^{4}(h_{i}(t))(v_{i}(t)-v_{K})\\ & & -g_{\mathrm{Na}}m_{\infty }^{3}(v_{i}(t))h_{i}(t)(v_{i}(t)-v_{\mathrm{Na}})\\ & & -g_{\mathrm{syn}}\sum_{j\neq i}s_{j}(t)(v_{i}(t)-v_{\mathrm{syn}})\\ \tau_{h}(v_{i}){\dot{h}}_{i}(t) & = & h_{\infty }(v_{i}(t))-h_{i}(t)\\ {\dot{s}}_{i}(t) & = & a(v_{i}(t-\tau_{d}))(1-s_{i}(t))-s_{i}(t)/\tau_{\mathrm{syn}} \end{array}$$

for i=1,…,N, where

$$\begin{array}{rcl} m_{\infty }(x) & = & \frac{0.1(x+40)/(1-e^{-(x+40)/10})}{0.1(x+40)/(1-e^{-(x+40)/10})+4e^{-(x+65)/18}}\\ h_{\infty }(x) & = & \frac{0.07e^{-(x+65)/20}}{0.07e^{-(x+65)/20}+1/(1+e^{-(x+35)/10})}\\ a(x) & = & 2/(1+e^{-x/(5(N-1))})\\ n(x) & = & \max\{0.801-1.03x,0\}\\ \tau_{h}(x) & = & {\left(0.07e^{-(x+65)/20}+1/(1+e^{-(x+35)/10})\right)}^{-1}. \end{array}$$

We consider the parameters $v_{\mathrm{Na}}=50$, $v_{K}=-77$, $v_{L}=-54.4$, $g_{\mathrm{Na}}=120$, $g_{K}=36$, $g_{L}=0.3$, c=1, I=10 and synaptic coupling parameters $g_{\mathrm{syn}}=0.08$, $v_{\mathrm{syn}}=0$, $\tau_{\mathrm{syn}}=20$.

**Fig. 2:**
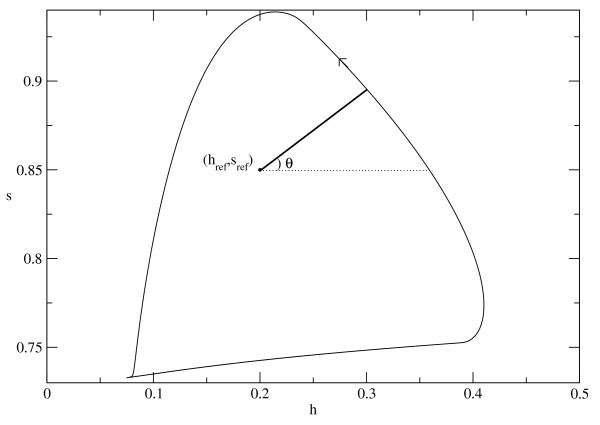
Oscillation of an uncoupled Hodgkin-Huxley type neuron (21) in the (h,s)-plane. The phase variable is estimated with respect to the reference point $\left(h_{\mathrm{ref}},s_{\mathrm{ref}})=(0.2,0.85\right)$.

The dynamics of this model is oscillatory for these parameter values. For the purpose of visualizing the dynamics, we define an approximate phase using a projection of the oscillation signal onto the h−s plane (see Figure 2). A reference point $\left(h_{\mathrm{ref}},s_{\mathrm{ref}})=(0.2,0.85\right)$ is chosen and the approximate phase is given as

$$\theta =\arctan\left[\frac{s-s_{\mathrm{ref}}}{h-h_{\mathrm{ref}}}\right].$$

For two different neurons we use the synchronization index

$$\rho_{ij}=\left|\frac{e^{i\theta_{i}}+e^{i\theta_{j}}}{2}\right|\leq 1$$

as a measure of their phase synchronization. The neurons *i* and *j* are completely phase synchronized when $\rho_{ij}=1$.

**Fig. 3:**
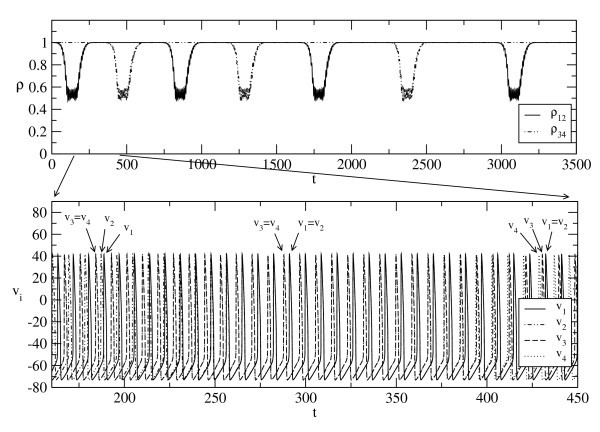
A solution of (21) for N=4 and $\tau_{d}=1.8$ approaching to a heteroclinic cycle between two cluster states with the same clustering {{1,2},{3,4}} but a different effective phase difference. In the upper graph, synchronization indices for neuron pairs (1,2) and (3,4) are plotted (after block averaging of size 1000), whereas in the lower graph a shorter time series of membrane voltages shows transitions between different synchronized clusters.

For N=4, a heteroclinic cycle exists as shown in Figure 3. This is a heteroclinic cycle between two saddle periodic orbits with the same clustering, that is {{1,2},{3,4}}. These saddle periodic orbits $x_{1}$ and $x_{2}$ form a heteroclinic cycle with a scheme

$$x_{1}\overset{I_{1}}{\to }x_{2}\overset{I_{2}}{\to }x_{1}$$

where

$$I_{1}:=\{v_{3}=v_{4},h_{3}=h_{4},s_{3}=s_{3}\},\;I_{2}:=\{v_{1}=v_{2},h_{1}=h_{2},s_{1}=s_{2}\}$$

and $x_{1},x_{2}\in I_{3}:=I_{1}\cap I_{2}$. Theorem 3 implies that as long as (a) the periodic orbits $x_{1}$ (resp. $x_{2}$) are hyperbolic, and (b) they are sinks when considered within the subspaces $I_{1}$ (resp. $I_{2}$) then the connection is robust. Condition (a) is generically satisfied. We have checked (b) using simulations by choosing different initial conditions for the dynamics reduced to $I_{1}$ and $I_{2}$. 

**Fig. 4:**
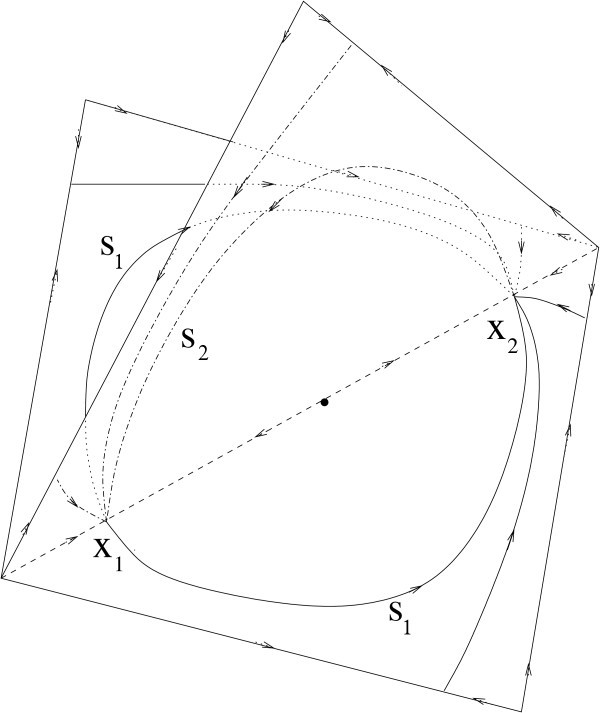
A robust heteroclinic cycle for four all-to-all coupled phase oscillator system analogous to the cycle found in Figure 3 for the Hodgkin-Huxley type system. The heteroclinic cycle consists of two saddle equilibria $x_{1}$ and $x_{2}$ and connections $s_{1}$ and $s_{2}$ on invariant subspaces. The invariant subspaces are embedded in a cube that represents a unit cell for the torus of phase difference space- in this representation the vertices represent in-phase solutions where all oscillators are synchronized. (Adapted from [22].)

 Coupled phase oscillators are used as simplified models for weakly coupled limit cycle oscillators, and one can find one-to-one correspondence between solutions if the coupling is weak enough [33]. In particular, the heteroclinic cycle depicted in Figure 3 corresponds in clustering type to a heteroclinic cycle found in [22] (see Figure 4) for a system of four globally coupled phase oscillators. The two saddle periodic orbits mentioned above correspond to the two saddle equilibria in Figure 4. 

**Fig. 5:**
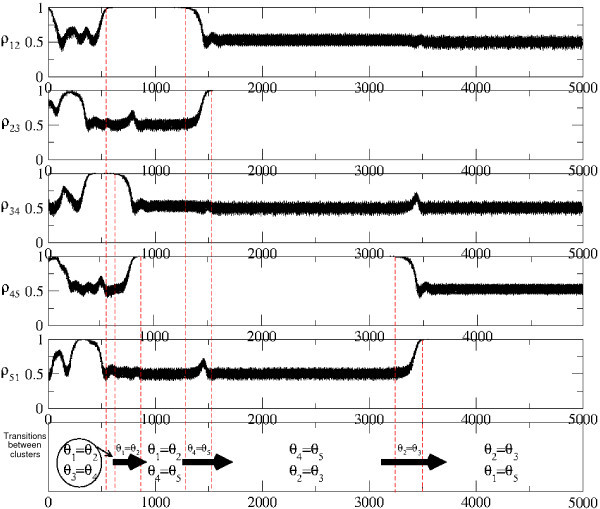
A trajectory of the system of N=5 neural oscillators (21) for $\tau_{d}=1.9$ approaching to a heteroclinic cycle between two clusters. In the first five graphs, five synchronization indices are plotted (after block averaging of size 1000) for different synchronized pairs, whereas in the last graph transitions between synchronized clusters are illustrated.

 For N=5, more complex heteroclinic cycles can appear as seen in Figure 5. *This is a heteroclinic cycle that connects different cluster states of type*(2,2,1). Note that the transition times between clusters are fixed but the duration of stay at each cluster gets longer and longer - a feature of attracting heteroclinic cycles. In the case of noisy systems, the dynamics switches from one cluster to another randomly around a graph of connections between symmetric cluster states [10]. 

 For globally coupled networks of N≥4 phase oscillators, robust heteroclinic cycles between cluster states have been found in [22,28,30]. Such robust heteroclinic cycles of coupled phase oscillators involve robust connections between saddle-type cluster states, where the robustness of the connections relies on them being contained within another nontrivial cluster state that corresponds to partially breaking the clusters and reforming them in a different way.

## 4 Discussion

In this paper we have introduced a testable criterion for robustness for a given cycle of heteroclinic connections within constrained settings - this test involves finding the connection scheme and then applying Theorem 1. We have attempted to clarify the similarity between winnerless competition dynamics in Lotka-Volterra systems as a special case of robust heteroclinic dynamics that respect some set of invariant subspaces in a connection scheme.

 Winnerless competition has previously been used to describe the competition of modes where at each mode a different neuron or neuron ensemble is active and other neurons or neuron ensembles remain inactive [8,34]. This type of competition relies on a stable robust heteroclinic cycle where robustness is due to the constraints on the individual dynamics of neurons. However, models where constraints are only on the coupling structure can admit a general phenomenon, namely robust heteroclinic cycles between cluster states. The model analyzed in Section 3.3 is an example with RHCs between cluster states. This dynamics relies on a stable robust heteroclinic cycle where robustness is due to the invariant subspaces forced by the coupling structure. In this case, the heteroclinic cycle connects saddle equilibria or saddle periodic orbits that represent different cluster states.

 We have not discussed the robustness of attraction properties of RHCs - mere existence of a RHC is not enough to guarantee that it will be an attractor, but we mention that as attraction properties are determined by open conditions on eigenvalues of the saddles (e.g. [1,24,35]), continuity of variation of the eigenvalues will guarantee that attractivity is also a robust property.

 For larger numbers of cells in symmetric or asymmetric arrays there may be very many such invariant subspaces, giving a wide range of possible robust heteroclinic cycles. Some of these are constructed in [15] for small numbers of coupled cells, but up to now there does not seem to be an easy way to explore which cycles are possible and which are not within any particular system. On the other hand, verifying that a particular heteroclinic cycle is, or is not, robust is a more tractable question that we address here. Note that which cycles exist may depend not just on having a valid connection scheme for some constrained set of vector fields, but also on the constraints not preventing the existence of the appropriate saddles or connections between them.

 Finally, we remark that there is evidence of metastable states in neural systems (e.g. [36-38]) that are supportive of the presence of approximate robust heteroclinic cycles. There are also suggestions that heteroclinic cycles may facilitate certain computational properties of neural systems - see for example [7,39,40].

## Competing interests

The authors declare that they have no competing interests.

## Footnotes

^1^We work within the class of continuously differentiable vector fields ($C^{1}$) to ensure, by the Hartman Grobman theorem [12], that hyperbolic equilibria are robust - this is a minimal requirement to discuss robustness of heteroclinic cycles.

^2^We take the subscripts modulo *p*.
